# Prognostic Variables of Younger-Aged Cervical Carcinoma Patients: A Retrospective Study

**DOI:** 10.1155/2021/5540165

**Published:** 2021-05-17

**Authors:** Emmanuel Kwateng Drokow, Lanlan Xu, Gloria Selorm Akpabla, Hafiz Abdul Waqas Ahmed, Juanjuan Song, Enyonam Adjoa Neku, Kai Sun

**Affiliations:** ^1^Department of Radiation Oncology, Zhengzhou University People's Hospital & Henan Provincial People's Hospital, Zhengzhou, Henan, China; ^2^Department of Hematology, Zhengzhou University People's Hospital & Henan Provincial People's Hospital, Zhengzhou, Henan, China; ^3^Department of Internal Medicine, Tianjin Medical University, Tianjin, China; ^4^Department of Pharmacy, Zhengzhou University, Zhengzhou, Henan, China

## Abstract

**Purpose:**

The prevalence of carcinoma of the cervix is increasing in younger women. This study aimed to evaluate the sociodemographic, pathological, and clinical features, prognosis, and treatment of women aged ≤35 years with carcinoma of the cervix (CC).

**Methods and Materials:**

We retrospectively analysed the clinical information of 352 younger women with carcinoma of the cervix aged ≤35 years at the Gynaecological Oncology Department of Zhengzhou University People's Hospital from April 2000 to January 2018. The overall survival was evaluated with the Kaplan–Meier model, and the log-ranked analysis was compared with the univariate analysis to determine prognostic survival-related risk factors. Cox Proportional Hazards analysis was further used in analysing parameters correlated with survival after univariate analysis. A *p* value <0.05 was considered statistically significant. SPSS version 23.0 was used for the data analysis.

**Results:**

The most frequent histopathological type observed in the selected 352 younger women was squamous cell carcinoma (SCC) (*n* = 221, 62.9%), adenocarcinoma (*n* = 125, 35.5%), and adenosquamous carcinoma (*n* = 6, 1.7%). The 5-year overall survival time was 80.5%. The prognostic risk factors discovered through univariate analysis were tumour stage (IA1-IIB vs. IIIA-IVA) (89.2% vs. 35.1%: *p* value = 0.002), histological type (SCC vs. non-SCC) (95.7% vs. 56.2%: *p* value = 0.001), surgical margin (negative vs. positive) (90.9% vs. 41.2%: *p* value = 0.001), and pelvic lymph node metastasis (no vs. yes) (93.4% vs. 39.2%: *p* value = .001). The Cox proportional hazards test demonstrated that lymph node metastases ([HR] = 2.924, 95% CI: 1.432–7.426; *p*=0.014), tumour stage IIIA-IVA ([HR] = 3.765, 95% CI: 1.398–9.765; *p*=0.016), and surgical margin ([HR] = 2.167, 95% CI: 1.987–9.554; *p*=0.019) were independent prognostic risk factors for overall survival in younger women with cervical carcinoma.

**Conclusion:**

In conclusion, the status of lymph node metastases, tumour stage, and surgical margin and the type of histopathology substantially influence the rate of survival.

## 1. Introduction

Cervical carcinoma (CC) is the fourth most common carcinoma in women worldwide. In 2018, about 570,000 new cases of cervical carcinoma were diagnosed, accounting for 6.6% of all female carcinomas [1]. Approximately 90% of cervical cancer deaths happen in middle- and low-income nations [2]. With comprehensive strategies such as effective screening, early diagnosis, prevention, and treatment programs, the high rate of mortality from cervical carcinoma worldwide could be minimized [3]. Also, human papillomavirus (HPV) vaccines have helped to decrease the risk of cervical carcinoma significantly [4]. In China, the annual incidence and mortality rate of cervical carcinoma is 106,430 and 47739, respectively; hence, CC is the sixth most common carcinoma among Chinese women and the third most frequent carcinoma in women between ages 15–44 years [5]. Nevertheless, the prevalence rate of CC in younger females is rising speedily. Patel et al. showed an annual 10.3% increment of CC incidence in women between the ages 20 to 29 years [6]. Li et al. showed that the incidence of CC in women ≤35 years was 16% [7]. Rutledge et al. and Lau et al. showed that younger women diagnosed with CC had a lower rate of survival and worse prognosis in comparison to elderly women [8, 9]. Nevertheless, some studies also reported no association between poor prognosis and age [10–12]. Davy et al. discovered that most younger women diagnosed with CC seem to have the adenocarcinoma histological type, hence the rise of this histological type in these patients, and this could be attributed to the fact that the human papillomavirus subtype associated with adenocarcinoma is more dominant in this category of women [13]. Again, the adenocarcinoma type has a poor prognosis when compared with squamous cell carcinoma (SCC). Furthermore, treatment complication arises due to fertility preservation or sparing in younger women; this, therefore, calls for a major in-depth knowledge of CC in this category of patients. Nevertheless, comprehensive literature and information related to younger women with CC are inadequate and limited. Also, the time frame for invasive carcinoma of the cervix to develop is above 5 years in about 30% of patients, but younger women with CC have been observed to have less than 5 years' history of cancer precursors or human papillomavirus infection [2]. We, therefore, hypothecate that younger women are prone to have an aggressive type of tumour. Furthermore, some socioepidemiological factors have been associated with cervical cancer development. We, therefore, retrospectively analyzed the clinicopathological, socioepidemiological, prognosis, and treatment of younger women with CC.

## 2. Materials and Methods

This study was conducted at the Gynaecological Oncology Department of our institution in China. Our retrospective study included younger women with CC treated at our institution from April 2000 to January 2018. Younger patients with pathologically confirmed carcinoma of the cervix were included. Our definition for younger women was patients ≤35 years. The medical records and database were reviewed to extract the clinicopathological information (number of sexual partners, age at menarche, age at first sexual intercourse, number of abortions, and family history of any gynaecological cancer), demographics (marital status, ethnicity, occupation, body mass index (BMI), and date of birth), symptoms (cervical polypoid mass, irregular vaginal bleeding, vagina discharges with odour, and postcoital bleeding), histological type, tumour stage, tumour size, type of surgical procedure, status of surgical margin, deep stromal invasion (DSI), lymphovascular space invasion (LVSI), parametrial involvement, and type of treatment received (chemotherapy, radiotherapy, and brachytherapy). Radical hysterectomy, conisation, and radical trachelectomy were the included surgical procedures. The 2009 International Federation of Gynaecology and Obstetrics staging system was used in staging patients. This study was approved by the ethics and institutional review board of Zhengzhou University and Zhengzhou University People's Hospital. The protocol of this study was according to the Helsinki Declaration. Confidentiality of patients' information was ensured in this study.

Follow-up of patients was carried out at the outpatient department or through telephone conversation up to April 2018. The overall survival period was described as the duration between the time of diagnosis to the last follow-up or the time of death. The overall survival (OS) was evaluated with the Kaplan—Meier model, and the log-ranked analysis was compared with the univariate analysis to determine prognostic survival-related risk factors. Cox Proportional Hazards analysis was further used in analysing parameters correlated with survival after univariate analysis. A *p* value <0.05 was considered statistically significant. SPSS version 23.0 was used for the data analysis.

## 3. Results

About 1676 women with cervical carcinoma were treated at the Zhengzhou University People's Hospital from April 2000 to January 2018, out of which 352 were patients younger or less than 35 years representing 21% of the entire carcinoma of the cervix patients diagnosed and treated at our centre. Tables 1 and 2 summarize the clinicopathological and demographic characteristics, respectively. The median age was 27 years during the period of diagnosis. The most frequent histopathological type observed in the selected 352 younger women was squamous cell carcinoma SCC (62.9%) followed by adenocarcinoma (35.5%) and adenosquamous carcinoma (1.7%). Prior to diagnosis, about 83.5% of the patients confirmed their involvement in sexual intercourse (activity), and the first sexual intercourse median age was 18 years. Also, over 70 (19.9%) patients had two or three multiple sex partners. Postcoital bleeding was the most common principal complaint presented to our institution by most patients (52.6%) followed by irregular vaginal bleeding (41.8%). None of the patients had their disease detected through cervical carcinoma screening. About 56.3% of the patients had stage II tumour, 23.8% had stage I tumour, and 15.8% and 4.0% had stage III and stage IV disease, respectively.

10 patients out of the 352 did not complete the entire treatment plan due to renal organ failure and financial issues. The conisation procedure was received by nineteen stage IA1 patients, and 24 stage IB1 patients with tumour size less than 20 mm underwent radical trachelectomy. Complete remission was achieved with radiochemotherapy in the five patients (5.3%) who had relapsed after radical trachelectomy. Furthermore, whole pelvic adjuvant radiotherapy was received by 100 patients following radical surgery with pathological postoperative risk factors (positive surgical margin, LVSI, and DSI), and over 80% of the patients received concomitant radiochemotherapy as the principal treatment. Postoperative whole pelvic radiation therapy was received by patients with one of these risk factors: tumour size >40 mm, positive lymph node metastasis, deep stromal invasion (DSI) > 1/2, lymphovascular space involvement (LVSI), parametrial involvement, and positive surgical margin. Extended field radiation therapy was only given after para-aortic lymph node metastasis or common iliac lymph node confirmation. The para-aortic LN and the whole pelvic were irradiated with 45–50.4 Gy in 25–28 fractions. High-dose-rate intracavitary brachytherapy with Iridium-192 was given to patients at a total dose of 24 Gy in 4-5 fractions. Lymphovascular space involvement (LVSI) was noticed in 56 patients, 69 patients had a deep stromal invasion, and 173 had lymph node metastasis.

The follow-up median time was 5.0 years (range: 1–17.5 years). Follow-up was performed at the outpatient department or through phone call conversation. Disease-free status was observed in 81.3% of the patients, while 12.7% experienced local relapse between the 3 months to 42 months after treatment completion. The total death recorded during the entire period was 13. The 5-year overall survival rate was 80.5%. The prognostic risk factors discovered through univariate analysis were tumour stage (1AI-IIB vs. IIIA-IVA) (89.2% vs. 35.1%: *p* value = 0.002), histological type (SCC vs. non-SCC) (95.7% vs. 56.2%: *p* value = 0.001), surgical margin (negative vs. positive) (90.9% vs. 41.2%: *p* value = 0.001), and pelvic lymph node metastasis (no vs. yes) (93.4% vs. 39.2%: *p* value = 0.001) (Table 3). The Cox proportional hazards test demonstrated that lymph node metastases ([HR] = 2.924, 95% CI: 1.432–7.426; *p*=0.014), tumour stage IIIA-IVA ([HR] = 3.765, 95% CI: 1.398–9.765; *p*=0.016), and surgical margin ([HR] = 2.167, 95% CI: 1.987–9.554; *p*=0.019) were independent prognostic risk factors for overall survival in younger women with cervical carcinoma (Table 4).

## 4. Discussion

Persistent infection with human papillomavirus leads to cervical carcinoma development. Invasive carcinoma of the cervix, precancerous changes, viral persistence, and infection with human papillomavirus are the four developmental stages in cervical carcinoma [5]. Generally, the time frame for invasive carcinoma of the cervix to develop is above 5 years in about 30% of patient's while changes in lesions normally start within five years of human papillomavirus infection [2]. Nevertheless, younger women with CC have been observed to have less than 5 years' history of cancer precursors or human papillomavirus infection. We, therefore, hypothecate that younger women are prone to have an aggressive type of tumour. Furthermore, most past investigations categorized younger women with CC as those ≤25 or ≤30 years, with evidence of heterogeneity and conflicting report on the prognosis for this ages' category [9–11]. Therefore, we concentrated on younger ladies with cervical malignant growth (≤35 years) for our research.

The histopathological subgroups distribution of CC in females aged ≤35 years varies from women (patient) of all age groups. Squamous cell carcinoma (65.5%) is the prevalent histopathological subtype common in women followed by adenocarcinoma subtype (28.5%) according to the SEER reports [14]. Nevertheless, the percentage of younger women having adenocarcinoma revealed in our study was higher than the percentage of adenocarcinoma among all ages group as reported by SEER, which further demonstrates that adenocarcinomas are more probable to be present in more younger patients. This affirms the rising of adenocarcinoma in younger women. Also, patients with adenocarcinoma purportedly have lower overall survival rate and worse prognosis in comparison to squamous cell carcinoma patients.

Preservation of fertility in younger women with cervical carcinoma is a significant factor. Radical trachelectomy which can be performed either robotically, vaginally, laparoscopically, and abdominally is the hallmark of conservative surgery. Radical trachelectomy has been excellently established in a number of research studies in terms of its feasibleness and safety [15–17]. In the present study, our data showed that radical trachelectomy was performed in 24 patients with the fundamental eligibility criteria to undertake this surgical procedure. Thus, clinical stage IA1 patients with lymphovascular space invasion and stage IB1 tumour less than 2 cm of any histopathological type (adenocarcinoma or SCC) had no clinical evidence of pelvic lymph node metastasis [18]. The obstetrical as well as the oncologic effects and results ought to be taken into thought in choosing radical trachelectomy since it is not the standard surgical procedure. Some previous studies reported that the less than 3% mortality rate and the less than 5% recurrence rate after radical trachelectomy were comparable to radical hysterectomy rates [19–24]. Also, a study by Bentivegna et al. showed that the rate of pregnancy after minimal invasive, abdominal, and vaginal radical trachelectomy was 65%, 57%, and 44%, respectively [25]. In addition, the range of live births rate after minimal invasive, abdominal, and vaginal radical trachelectomy in Bentivegna's study was between 67–78%. In our study, complete remission was achieved with radiochemotherapy in the five patients (5.3%) who had relapsed after radical trachelectomy. Also, our rate of live birth was 69% which is comparable to the live birth rate published by some past studies. Hence, radical trachelectomy ought to be viewed as a discretionary surgical procedure in the management of younger women with early cervical carcinoma with fertility preservation in mind.

The 5-year overall survival rate for all women with carcinoma of the cervix varies from 56–78% [26–29]. The 5-year OS rate is 78% for women aged 50 years or below. Yan et al. reported that the 5-year OS rate for CC patients was 81.9% [30]. The 5-year OS rate in our study for younger women with CC aged ≤35 years was 80.5% which demonstrates that the survival of younger women with CC is not worse when compared to elderly women. Additionally, our study also showed that the OS rate varies depending on the histopathological subgroup of cervical carcinoma. Furthermore, a significant *p* value was achieved in the OS rate between non-SCC and SCC of the cervix (56.2% vs. 95.7%: *p* value = 0.001). These findings suggest that the histopathological type influences the rate of survival and that women with SCC histopathological type have a better prognosis than non-SCC type, indicating consistency with past research. In addition, based on univariate analysis, we discovered that the status of lymph nodes metastases, tumour stage, surgical margin, and the type of histopathology substantially influenced the rate of survival. Nevertheless, no significant *p* value was observed in the histopathological subtype during multivariate analysis.

Selection bias is one of the limitations in our study due to the scarcity of cervical carcinoma in women aged ≤35 years. Also, a reference group of elderly women was not included in this study; hence, comparison of various factors was carried out with past published studies. Lastly, there are other risk factors such as performance status, tumour grade, haemoglobin level, and type of HPV that can affect the prognosis and survival of patients with cervical cancer. Nevertheless, most of the information for some of these confounding factors was not available resulting in selection bias. Our study, however, is the largest study on women aged ≤35 years with carcinoma of the cervix. In addition, we were able to discover factors affecting prognosis in this age category.

## 5. Conclusions

In conclusion, the overall prognosis for younger women with CC is comparable to that of elderly patients. Nevertheless, the rate of survival appears to vary extensively depending on the histopathological classification; thus, the rate of survival for SCC patients is higher than that of non-SCC patients. In addition, much focus should be given to patients with lymph node metastasis, higher tumour stage, and positive surgical margin since these variables predict bad prognosis.

## Figures and Tables

**Table 1:** Clinical characteristics of patients.

Clinical characteristics	*N* = (352)	Percentage (%)
*Histopathology*		
Squamous cell carcinoma	221	62.9
Adenocarcinoma	125	35.5
Adenosquamous	6	1.7

*Tumour stage*		
I	84	23.8
II	198	56.3
III	56	15.8
IV	14	4.0

*Symptoms*		
Irregular vaginal bleeding	147	41.8
Postcoital bleeding	185	52.6
Cervical polypoid mass	20	5.7

*History of sexual behavior*		
Yes	294	83.5
No	58	16.5

*Surgery*		
Radical hysterectomy	74	21.0
Radical trachelectomy	24	6.8
Conisation	19	5.4

*Differentiation*		
Well differentiated	175	49.7
Moderately	83	23.6
Poorly	67	19.0
Unknown	27	7.7

*Adjuvant therapy*		
Radiation therapy	100	28.4
Concurrent chemoradiotherapy	224	63.6
No	28	8.0

**Table 2:** Demographical characteristics of patients.

Demographical features	*N* = (320)	Percentage (%)
*Age*		
Range	15–35	
Median	27	

*Ethnicity*		
Han	345	98.0
Minor	7	2.0

*Socioeconomic status*		
Middle income	69	19.6
Low income	283	80.4

*Marital status*		
Married	125	35.5
Unmarried	227	64.5

**Table 3:** Univariate analysis outcomes.

Factors	5-year OS	*p* value
*Tumour stage*		
IA1-IIB	89.2%	0.001
IIIA-IVA	35.1%	

*Histological type*		
SCC	95.7%	0.002
Non-SCC	56.2%	

*PLNM*		
Yes	39.2%	0.001
No	93.4%	

*Differentiation*		
Well differentiated	75.1%	0.710
Moderately	71.4%	
Poorly	68.2	

*Surgical margin status*		
Positive	41.2%	0.001
Negative	90.9%	

OS = overall survival, SCC = squamous cell carcinoma, PLNM = pelvic lymph node metastasis.

**Table 4:** Multivariate analysis outcomes.

Prognostic factors	HR [95%CI]	*p* value
PLNM	2.924 [1.432–7.426]	0.014
Tumour stage	3.765 [1.398–9.765]	0.016
Surgical margin	2.167 [1.987–9.554]	0.019

PLNM = pelvic lymph node metastasis, HR = hazard ratio, CI = confidence intervals.

## Data Availability

The data analysed in the current study are available with the corresponding author and can be released on reasonable request.
